# Why Psychiatry Needs 3,4-Methylenedioxymethamphetamine: A Child Psychiatrist’s Perspective

**DOI:** 10.1007/s13311-017-0531-1

**Published:** 2017-05-05

**Authors:** Ben Sessa

**Affiliations:** Psychopharmacology Department, Department of Medicine, Imperial College London University, Burlington Danes Building, 160 Du Cane Road, London, W12 0NN UK

**Keywords:** MDMA, PTSD, Psychotherapy, Trauma, Psychedelics, addictions

## Abstract

Since the late 1980s the psychoactive drug 3,4-methylenedioxymethamphetamine (MDMA) has had a well-known history as the recreationally used drug ecstasy. What is less well known by the public is that MDMA started its life as a therapeutic agent and that in recent years an increasing amount of clinical research has been undertaken to revisit the drug’s medical potential. MDMA has unique pharmacological properties that translate well to its proposed agent to assist trauma-focused psychotherapy. Psychological trauma—especially that which arises early in life from child abuse—underpins many chronic adult mental disorders, including addictions. Several studies of recent years have investigated the potential role of MDMA-assisted psychotherapy as a treatment for post-traumatic stress disorder, with ongoing plans to see MDMA therapy licensed and approved within the next 5 years. Issues of safety and controversy frequently surround this research, owing to MDMA’s often negative media-driven bias. However, accurate examination of the relative risks and benefits of clinical MDMA—in contrast to the recreational use of ecstasy—must be considered when assessing its potential benefits and the merits of future research. In this review, the author describes these potential benefits and explores the relatives risks of MDMA-assisted psychotherapy in the context of his experience as a child and adolescent psychiatrist, having seen the relative limitations of current pharmacotherapies and psychotherapies for treating complex post-traumatic stress disorder arising from child abuse.

**Electronic supplementary material:**

The online version of this article (doi:10.1007/s13311-017-0531-1) contains supplementary material, which is available to authorized users.

## Introduction: From Child Abuse and Mental Disorder to Addictions and 3,4-Methylenedioxymethamphetamine

I am a child and adolescent psychiatrist, who also works with adults with addictions. My adult patients in their 20s, 30s, and beyond with unremitting depression, anxiety, post-traumatic stress disorder (PTSD), and substance abuse are sadly the same type of patients that I also care for as abused children. I believe it is a travesty that after 100 years of modern psychiatry we are not better at managing the roots of lifelong trauma-based disorders. There is a pervasive sense of learned helplessness within psychiatry. We dare not use the word “cure” when it comes to trauma, but rather have become expert at seeing our patients as palliative cases who present in their adolescence and keep coming back to psychiatry for the rest of their lives. We paper over the cracks of their pain with the prescribing of lifelong maintenance drugs; medicines that, if we are lucky, keep symptoms at bay but never get to the root cause of their pain: that early childhood experience of trauma. The lack of efficacy of traditional treatments—both pharmacological and psychotherapeutic—and the recognition that something must be done for these patients, has brought me to the door of 3,4-methylenedioxymethamphetamine (MDMA)-assisted psychotherapy. As a pharmacological agent MDMA has multiple complex and idiosyncratic modes of action. And in combination with psychotherapy the medicine provides the capacity to hold the traumatized patient in a state of emotional security, providing a state of empathic self-reflection in which, for the first time in their lives, they can be *with* their traumatic memories without being overwhelmed by the powerful negative affect that usually accompanies recall of their most frightening thoughts.

## The Effect of Trauma on the Developing Child’s Brain

During those early years, the quality of a child’s attachment relationship with their primary caregiver becomes a blueprint of emotional containment that is carried into adulthood; forming the basis of intimate relationships. It is not only the ‘social services radar’ forms of abuse—physical and sexual abuse—that can disrupt this development. Emotional abuse and neglect, perhaps seen as less damaging by many, also inflicts considerable psychological damage; leaving a survivor feeling unwanted and worthless. Disruption to the early attachment relationship leaves a person vulnerable to PTSD and the other anxiety-based disorders, reducing their capacity to make and form relationships and causing lasting feelings of low self-esteem [1].

Being raised in a home environment of chaos and fear causes the sustained release of stress hormones, including cortisol, which results in the development of an exaggerated sense of fear, driven by the amygdala, and an attenuated ability to exercise a prefrontal cortex (PFC)-mediated fear extinction response [2]. PTSD is characterized by this imbalance in the PFC–amygdala dynamic [3], and seeing no way out of this cycle of fear many patients turn to substance misuse to blunt their feelings [4], and, similarly, rates of self-harm and completed suicide are high [5].

## Treating Trauma-Related Disorders, Including Addictions

As medical doctors, with traditional education and methodical approaches, we are generally trained to recognize, categorize, and adjust the pathology before us. But there is no established single drug or psychotherapy treatment that gets to the root cause of trauma. Rather, we manage the individual symptoms as they emerge. If the patient presents with low mood, give an antidepressant; if they complain of poor sleep, give a hypnotic; if their moods fluctuate wildly, prescribe a mood stabilizer; and if their hypervigilance—one of the core features of PTSD—progresses into frank psychosis then prescribe an antipsychotic [6]. These drug therapies, whilst undoubtedly beneficial at relieving symptoms in many cases of PTSD, are only partially effective. There remains a staggering 50% treatment resistance for half of PTSD sufferers [7]. Similarly, the wide range of psychotherapies available to treat trauma, from cognitive behavioral therapy, to dialectical behavioral therapy to eye-movement and desensitization reprocessing, are only partially effective in some of the cases. For many, the chronicity of the disorder and the intense cluster of avoidance behaviors makes accessing those crucial traumatic memories a significant barrier to progress. By the time patients come to the attention of services, often already in the throes of addiction, they are so well-defended that thought of “being with those memories” to address and resolve them, simply triggers the knee-jerk response to fall into a dissociative state of self-preservation.

## How MDMA Psychotherapy Works for Treating Trauma

MDMA is a ring-substituted phenethylamine, whose pharmacokinetics and pharmacodynamics have been well studied in humans. It exerts is effects primarily through promoting raised levels of monoamine neurotransmitters in the brain, especially serotonin, but other mechanisms are also involved. Table 1 describes the receptor profile of the drug and relates the effects to its efficacy as a psychotherapeutic agent. Increased activity at 5-hydroxytryptamine (HT)_1A_ and 5-HT_1B_ receptors reduces feelings of depression and anxiety, reduces the amygdala fear response, and increases levels of self-confidence [9]. Furthermore, the effect of raised serotonin at 5-HT_2A_ receptors provides alterations in the perceptions of meanings and facilitates new ways of thinking about old experiences [10]. The effect of increased dopamine and noradrenaline is to raise levels of arousal and awareness, which increases sense of readiness and improves recall of state-dependent memories of stressful events [11]. Alongside this increase in arousal, which can motivate a user to engage in therapy, there is also a paradoxical increase in relaxation, which counters hypervigilance effects, mediated by MDMA’s action at alpha-2 receptors [12]. MDMA has also been shown to facilitate the release of oxytocin—the hormone associated with early infantile bonding and increased levels of empathy and closeness [13]. These well-documented effects of MDMA used clinically give rise to its description as an “empathogen” or “entactogen” [14].

**Table 1:** Relating 3,4-methylenedioxymethamphetamine’s (MDMA) psychotherapeutic effects to its unique receptor profile [8]

Receptors or brain region involved	MDMA effects	How effects relate to treatment of PTSD	Neurobiological correlates	
Serotonin	Reduces depression and anxiety	Provides patient with an experience of positive mood and reduced anxiety in increased engagement	Release of presynaptic 5-hydroxytryptamine (5-HT) at 5-HT_1A_ and 5-HT_1B_ receptors	
	Stimulates alterations in the perceptions of meaning	Opportunity to see old problems in a new light	Optimum level of arousal	Increased activity at the 5-HT_2A_ receptors
Dopamine and norepinephrine	Raises levels of arousal	Stimulating effect increases motivation to engage in therapy		Release of dopamine and noradrenaline
Alpha-2 adrenoreceptors	Increases relaxation	Reduces hypervigilance associated with PTSD		Increased alpha 2-adrenoceptor activity
Hormonal effects	Improves fear extinction learning	Allows reflection on traumatic memories during psychotherapy without being overwhelmed	Release of noradrenaline and cortisol	
	Increases emotional attachment and feelings of trust and empathy	Improved relationship between patient and therapist. Capacity to reflect on traumatic memories	Multiple factors, including release of oxytocin	
	More likely to use words relating to friendship, and intimacy	Generate discussion about wider aspects of the patient’s social and emotional relationships		
	Reduced social exclusion phenomena	Opportunity to reflect upon patients’ wider social functioning		
Regional brain changes	Improved detection of happy faces and reduced detection of negative faces	Enhances levels of shared empathy and prosocial functioning	Increased PFC activation and decreased amygdala fear response	
	Reduced subjective fear response on recall of negative memories	Opportunity to reflect upon painful memories of trauma during psychotherapy	Decreased cerebral blood flow in the right amygdala and hippocampus	

PTSD = post-traumatic stress disorder; PFC = prefrontal cortex

As a psychotropic drug, MDMA provides a remarkably consistent, subjectively positive, experience. However, when employed as a tool to treat trauma, the experience is nevertheless challenging, as, despite experiencing the positive effects of MDMA, recall of traumatic memories is often difficult. It is shorter acting than most classical psychedelic drugs [2–5 h duration compared with 4–8 h for psilocybin and 8–12 h for lysergic acid diethylamide (LSD)], it produces fewer perceptual alterations and is therefore well tolerated. MDMA produces elevated mood, increased sociability and feelings of closeness to others, greater compassion, feelings of sociability, closeness, and increased empathy for others and themselves [15, 16]. MDMA’s effect at reducing activity in the amygdala results in users experiencing a reduced fear response and greater activity in the reward pathways, suggesting less reactivity to anger and improved prosocial behavior [17, 18]. Because of the totality of MDMA’s receptor actions and associated psychological effects, providing the optimum level of arousal [19] MDMA has been dubbed “the perfect drug for psychotherapy” [20]. In combination, these effects allow the MDMA user, when the drug is taken within the security of a facilitating psychotherapeutic relationship, to *be with* their memories of trauma without being overwhelmed by negative affect. Within this psychotherapeutic environment the traumatized patient can begin the process of addressing their painful memories.

## Is MDMA Safe or Dangerous?

Despite the popular media’s tendency to polarize and simplify MDMA’s safety profile based on recreational ecstasy use, in clinical medicine we have a more sophisticated way of looking at clinical possibility than simply asking if an intervention is safe or dangerous. After all, are knives safe? Are cars dangerous? The answer is always “It depends”. Rather, we ask the question: What are the *relative risks versus* the *relative benefits* of using *this intervention* at *this time* in *this patient*? All medical interventions from sticking plasters to cancer chemotherapy are both invasive and beneficial at the same time. So, rather than making inferences about the usefulness of MDMA therapy based on erroneous preconceptions about recreational ecstasy use, we must dispassionately apply the principles of evidence-based clinical governance.

When I first started writing and presenting on MDMA I would spend a significant part of my time addressing and defending MDMA therapy in response to audiences’ questions about recreational ecstasy use. But I recognize now that was folly. Clinical MDMA and recreational ecstasy use share few comparable features; whether in terms of the purity of the drug, the way it is administered, or the safety measures associated with the screening and monitoring of selected participants. After > 1600 doses of the drug being administered in a clinical setting there has been only one report of a brief, self-limiting serious adverse event [21]. Clinical MDMA is *not* recreational ecstasy.

However, even when we do look at recreational ecstasy we see very low rates of morbidity and mortality. In the UK, an estimated 750,000 doses of ecstasy have been consumed *every weekend* for the last 25 years. Yet rates of harm remain very low indeed. A study in 2003 looked at UK coroners’ reports of deaths attributed, in part, to ecstasy between 1997 and 2000 and found 81 such deaths [22]. However, only 7% of those 81 deaths involved MDMA alone. That is 6 deaths in 3 years, after some 120 million ecstasy tablets were consumed by over 1 million people.

MDMA may be acutely toxic through hyperthermia [23] or through hyponatremia [24]. The former may occur through prolonged physical exertion in a hot environment, combined with dehydration due to not consuming enough water. High temperature has also been demonstrated to further exacerbate the risk of longer-term neurotoxicity [25]. The second cause of acute toxicity is ecstasy-induced hyponatremia, in which vulnerable individuals with a genetic predisposition experience an impairment of the kidney’s normal water homeostasis mechanism. This can be further exacerbated by excessive water consumption, which can occur, ironically, because users may be overvigilant about the risk of dehydration. These harms, when observed through recreational use, highlight the recognized phenomenon that it is the prohibition of MDMA, as with other illicit drugs, that has an important bearing on the riskiness of its use. Indeed, it is arguable that the risk-averse approach of governments, driven by the scare-mongering popular media and vice versa, contributes significantly to the harms associated with recreational ecstasy use, and, as is well documented, making researching the compound unnecessarily expensive and time-consuming [26].

## Postecstasy Come Down: Is it Due to MDMA?

When presenting on MDMA science, people often ask about the “mid-week blues” phenomenon. There is a well-documented narrative amongst recreational ecstasy/MDMA users community about the infamous “blue Monday” and “black Tuesday”. User forums (such as Bluelight, http://www.bluelight.org) frequently discuss the phenomenon, describing low mood, irritability, and fatigue for several days after using the drug recreationally. But when ecstasy is taken recreationally users frequently take the drug at night, thereby missing a night’s sleep; they dance; often use other drugs, including alcohol; and often go without food. Studies prospectively assessing recreational ecstasy users have demonstrated such effects but concluded that sleep disturbance and concomitant drug use, including alcohol, explain the findings [27–30]. An interesting study on a unique group of recreational MDMA users, Mormons in the USA, who use no other drugs, including alcohol, demonstrated no significant neurocognitive or mood deficits after taking the drug recreationally (T. Passie, personal communication, 2017) [31].

Many users anecdotally cite the “serotonin depletion” argument for their symptoms, which hypothetically sounds like a reasonable theory to explain post-MDMA low affect. But there is little data for this hypothesis. There have not been any studies looking at serotonin in humans in the days after MDMA. Franz Vollenweider, MDMA researcher for the Heffter Institute in Switzerland, has done the only clinical study of MDMA and the serotonin transporter, SERT, in humans and found no change in SERT binding after 1 month [32].

This issue of “mid-week blues” highlights, again, the folly of making inferences about the relative effects and risks of MDMA based on the anecdotal reports of recreational ecstasy users. When pure clinical MDMA is delivered during the day, with no missed sleep and a good evening meal afterwards, as is the pattern of dosing when it is used clinically, do significant numbers of people complain of postsession low affect? Or are most cases of Monday blues simply an artifact of recreational use?

We turn to clinical studies with MDMA to explore this question. George Greer conducted open-label studies with subjects receiving group and individual therapies with MDMA in the early 1980s before the drug was banned. Participants took pure MDMA, did not miss out on sleep, and did not dance. Most people reported low energy for a day or so after their daytime sessions, but there was no significant mood disturbance (G. Greer, personal communication, 2017) [33].

In more recent double-blind controlled studies exploring MDMA-assisted psychotherapy for treatment-resistant PTSD, both the MDMA and placebo groups described fatigue, anxiety, low mood, headache, and nausea in the week after experimental sessions, with low mood being slightly more frequent in the *placebo* group [34]. These data do not support a clear postsession syndrome attributable only to the pharmacological effects of MDMA. Furthermore, Mithoefer (personal communication, 2017) reflects whether recreational ecstasy-induced mid-week blues may highlight the presence of unprocessed psychological process that is left stirred-up, but unresolved, without a therapeutic set and setting. However, looking more closely at the pooled outcome data of all the recent Multidisciplinary Association for Psychedelic Studies (MAPS)-sponsored MDMA/PTSD studies in which mood was assessed using the self-report Beck Depression Inventory measure, > 30% of people reported mood changes in the days after taking clinical MDMA [21]. However, it is not clear whether the reported “depressed mood” is confounded by fatigue, a commonly reported effect often experienced as low mood (I. Jerome, personal communication, 2017).

In our 2 UK-based forthcoming MDMA studies we will be examining mood and affect for 7 days after MDMA and placebo sessions, which will add further data to this interesting aspect of the effects of MDMA. At this stage, given the large numbers of ecstasy users that report mid-week blues, despite the presence of confounding factors when the drug is used recreationally, there likely *is* a mild effect on mood post-MDMA in a certain minority of users. This may be due to serotonin depletion, but much more robust, controlled research needs to be done on the phenomenon. A particularly interesting study would be to test affect post-MDMA in the following prospective manner, where groups A/B represent *clinical MDMA* and groups C/D represent *recreational use of ecstasy*: 1) *group A*: Take MDMA at 10 a.m., no exercise, sleep normally that night; 2) *group B*: take placebo at 10 a.m., no exercise, sleep normally that night; 3) *group C*: take MDMA at 10 p.m., exercise, stay up all night, missing out on sleep; 4) *group D*: take placebo at 10 p.m., exercise, stay up all night, missing out on sleep.

Further variations of such a study could also test the effects of missing out on food and taking concomitant drugs including alcohol. And finally, on this issue, such a study could also include groups to test the much-reported phenomenon of 5-hydroxytryptophan supplements. Anecdotally many recreational ecstasy users take 5-hydroxytryptophan as a reliever of post-MDMA low affect but the effect remains entirely untested in controlled experimental conditions with pure MDMA.

## Contemporary Clinical Studies with MDMA-Assisted Psychotherapy

Before MDMA was banned in the mid-1980s psychiatrist George Greer, together with his wife and psychiatric nurse co-therapist, Requa Tolbert, carried out a series of uncontrolled case studies describing the use of MDMA with couples or in groups. They describe the drug as a tool to enhance communication and closeness [33], outlined recommended conditions for its use as therapeutic agent [35], and they described some of the subjective effects associated with its use [36].

Since the drug was banned, becoming a Schedule One substance, in 1986, research has been slow and challenging. Between 1988 and 1993 the Swiss Medical Society for Psycholytic Therapy conducted individual and group psychotherapy with MDMA. Over 100 patients with a wide range of psychiatric problems (personality, adjustment, affective and eating disorders and addiction, psychosis, and sexual deviation problems) each received an average of 8 drug-assisted sessions with MDMA or LSD. Doses of MDMA averaged 125 mg. After treatment and 19 months of follow-up > 90% of patients had experienced good or slight improvement [37]. However, in the wake of growing recreational use of ecstasy the Swiss authorities shut the project down in 1993—despite calls from the Swiss therapists to plan a more methodologically robust study. In 2009, German psychiatrist, Friederike Meckel, who had trained with the Swiss Medical Society for Psycholytic Therapy, was briefly imprisoned after being caught continuing to practice underground therapy with MDMA, LSD, and 2C-B. Although the outcomes of Dr. Meckel’s therapy were not quantitatively measured, the qualitative reports of success with her patients were strongly in favor of the benefits of psychedelic drug-assisted psychotherapy. This incident shed some light on the previously unknown scale of underground psychedelic therapy occurring in Europe [38].

In recent decades, MAPS has lead the way in global MDMA/PTSD studies and has amassed a pool of data from phase II studies in USA, Switzerland, Israel, and Canada. In their first pilot study, published by Michael Mithoefer in 2011, 20 patients with chronic PTSD, refractory to both psychotherapy and psychopharmacology, received either 2 or 3 sessions of placebo-controlled MDMA-assisted psychotherapy alongside a course of preparatory and follow-up nondrug psychotherapy. Using the Clinician-Administered PTSD Scale (CAPS) as a primary outcome measure, Mithoefer showed that at the 2 and 12-month follow-up 83% of the experimental group no longer met the criteria for PTSD compared with just 25% of the patients in the placebo group. There were no drug-related serious adverse events, adverse neurocognitive effects, or clinically significant physiological events [34]. The cohort was subsequently followed-up for an average of 42 months after the initial single course of MDMA-assisted psychotherapy. And in 2013 results of that long-term follow-up study showed that rates of remission were largely maintained, without having any further doses of MDMA since the original study. The long-term follow-up showed that 17 of the 19 subjects had sustained remission by self-report; however, by the long-term follow-up CAPS scores were obtained for only 16 of the 19, so the actual percentage of sustained remission on CAPS is not certain [39]. Also in 2013, psychiatrist Peter Oehen published the results of his MAPS-sponsored MDMA psychotherapy study for treatment-resistant PTSD. Together with co-therapist, Verena Widmer, Oehen’s was a smaller study than Mithoefer’s and although there was a definite trend in the direction of MDMA therapy being superior to placebo, at first sight the statistics failed to demonstrate a significant reduction in CAPS for the experimental subjects [40]. However, Henri Chabrol of Toulouse University looked at the data again using effect size as a measure and concluded that results *were* indicative of MDMA psychotherapy providing substantial improvements for treatment-resistant PTSD [41].

There have been several other clinical MDMA studies in recent years that have failed to progress in the context of the multiple complexities associated with carrying out MDMA research in today’s political climate. These include the Spanish MDMA for PTSD study, run by José Carlos Bouso in Madrid, which started in 1999 but was shut down in 2002 as a result of political pressure. Another MDMA study run by John Halpern at Harvard University planned to explore the role for MDMA to treat Anxiety Secondary to Advanced Stage Cancer. It began recruiting in 2004 but was subsequently closed owing to enrolment challenges. Another MAPS-sponsored MDMA study, in Jordan, lead by Nasser Shuriquie, has been delayed awaiting Jordanian approval to begin the project. Similarly, in Australia a team lead by Stuart Saker and Fiona MacKenzie from Sydney has since met with authoritarian resistance and failed to progress. It is also worthy of note that Dr. Charles Grob’s study of psilocybin therapy for end-stage cancer patients [42] was originally planning to use MDMA. However, Grob had concerns surrounding the potential cardiovascular risks of MDMA in a medically frail group of patients. He was also concerned that in the context of increasing negative publicity regarding growing recreational ecstasy use, there was emerging a perceived over-sensationalizing of so-called "MDMA neurotoxicity”, which might preclude clinical MDMA protocols getting a fair hearing. Grob subsequently switched to psilocybin for this study.

## The Future for MDMA Clinical Research: Training MDMA Therapists

In November 2016 MAPS received approval from the Food and Drug Administration to go ahead with phase III studies, and now plan multicenter, multinational studies, including plans for several sites in the UK and Europe. Pending the starting, and eventual outcome, of these studies, MAPS states 2021 as a potential date when MDMA could be licensed for prescribed practice. In which case the field will need many new MDMA therapists in the next few years. MAPS has subsequently begun a program of therapist training, with teams based in Boulder, Colorado, and Charleston, South Carolina. Training entails various stages, which include reading the MAPS manual on how to deliver MDMA therapy for PTSD, completing the MAPS online training modules, undertaking a week-long video training watching many hours of video footage of patients undergoing MDMA therapy, taking part in role play, discussion and nondrug experiential breath-work sessions, and, finally, undergoing an MDMA and a placebo therapy session with trained guides. While it is relatively easy to organize large-scale video training and discussion groups, is a bottle-neck when it comes to getting the experiential training with MDMA. To date, only a small handful of people around the world have completed the full training.

## The Cardiff Functional Magnetic Resonance Imaging MDMA Study

pt?>Whilst the forthcoming MAPS phase III studies are set to develop a firm basis for using MDMA therapy for PTSD, it is noted that the acute effects of MDMA in subjects with PTSD have never been investigated using neuroimaging techniques. In view of this, I am planning a study at Cardiff University, sponsored partially by MAPS and the Beckley Foundation, to start later in 2017. The aims are to define and explore the neural correlates of MDMA in subjects with PTSD. This will involve imaging cerebral blood flow and brain activity under task-free resting conditions and measuring brain activations in response to 2 experimental paradigms and 1 postscan measure of cognitive and emotional empathy using a randomized, double-blind, placebo-controlled, crossover method. Subjects with treatment-resistant PTSD will take MDMA (125 mg) and placebo in 2 experimental conditions separated by 2 weeks. This is an exploratory study and no additional psychotherapy will be offered. However, owing to the novel nature of the MDMA experience, the study psychiatrist and a female co-sitter, both trained in MDMA therapy, will sit with the participants in a supportive, facilitative environment until the acute effects have dissipated. We hypothesize that in the MDMA session there will be reduced amygdala and increased PFC activity that will positively correlate with subjective ratings of the intensity of the MDMA experience.

## Is There a Role for MDMA Therapy for Addictions?

Despite the rich history of classical psychedelics to treat addictions, MDMA has never been studied for this proposed use. There have been scant animal studies on the subject, with just 1 study showing an attenuation of alcohol use in rats after administration with MDMA [43]. In our proposed UK-based study, we postulate that MDMA-assisted psychotherapy could be useful in treating alcohol use disorder through its capacity to enhance the psychotherapeutic process or indirectly through augmenting the treatment of comorbid psychological conditions commonly associated with alcohol dependence [44]. In Greers’ report in the mid-1980s MDMA users, who were not specifically from a substance misuse population, reported reduced substance use after at least 1 session of MDMA-assisted psychotherapy, with 14 of 29 patients reporting a decreased desire for substances such as alcohol, cannabis, and caffeine [33]. A recent study in a naturalistic setting with recreational MDMA users has shown enhanced prosocial attitudes towards the self, especially for patients with pre-existing histories of trauma [45]. This capacity for MDMA to increase feelings of empathy and compassion for the self and others may also contribute to improved self-awareness and subsequently reduce the denial of alcohol abuse. Similarly, Mithoefer (personal communication, 2017) described MDMA’s capacity to “make yourself present in the moment”, which is a core concept of mindfulness. And in recent years mindfulness techniques, which encourage awareness and acceptance of thoughts, promote nonjudgmental acceptance of moment-to-moment thoughts can therefore help the alcohol addict to interrupt the tendency to react to cravings with alcohol use [46–48].

But will MDMA for addictions work? Drug-assisted psychotherapies with the “classical” psychedelic compounds, such as LSD and psilocybin, have found that the subjective mystical/spiritual effects of the psychedelic experience are strongly associated with maintained abstinence from substance use [49–51]. However, there is a subjective spiritual/mystical experience associated with MDMA use, albeit generally less so than with “classicals” [52, 53]. Furthermore, not all patients are able to tolerate the perceptually disturbing effects of the classical psychedelic experience, or even the *idea* of a treatment involving ‘classicals’, and compliance is a critical part of addiction therapy. Therefore, MDMA offers an alternative opportunity for enhanced psychotherapy.

This year in Bristol, UK, we will use an open-label design and aim to recruit 20 patients with alcohol use disorder who have completed a successful community detoxification to a 10-week program consisting of 2 sessions of MDMA-assisted therapy and 8 sessions of non-MDMA-assisted therapy. The study will test if MDMA-assisted psychotherapy is feasible in this patient group, if it is tolerated and if it can be delivered safely in a clinical setting, offering patients a potential new approach to this devastating, chronic, and relapsing condition.

## The Importance of Tackling Misinformation and Stigma

Whenever a young person dies from a drug-related event involving ecstasy it tends to make the front page, which exaggerates the relative risks of ecstasy—and bears little resemblance to clinical MDMA. Annually in the UK there are around 9000 alcohol-related [54] and 80,000 tobacco-related deaths [55]. Nevertheless, the tremendous public relations “success” of the sustained War on Drugs continues to contribute to many negatively biased attitudes towards MDMA and other psychedelic substances, whilst disregarding the great harms done by legal drugs.

By way of an illustration of the fears surrounding psychedelic medicine, when I was presenting at a conference in 2015 to the Royal College of Psychiatrists about the benefits of psychedelics, a concerned emergency room doctor in the audience stuck up his hand and expressed his displeasure at hearing me suggest that LSD was relatively safe. He described what he considered a dangerous incident, in which a young person came into the emergency room having taken *17* tabs of acid. The young person had removed his clothes and was performing somersaults in the hospital corridors. I asked the doctor whether the young person required admission to hospital or subsequent referral to psychiatric services. “No”, he said, “After several hours of monitoring he was allowed home under the supervision of his friends and he required no further follow-up”. I put it to the challenging doctor what might have been the scenario had someone attended his department having overdosed on 17 g of cocaine, 17 g of heroin, or, for that matter, 17 bottles of wine? The doctor acknowledged that under those scenarios such a person would have ended up in intensive care or could have easily died. “So,” I said to the audience member, “here is a young person who took 17 times the recommended dose of a substance and the only pathology was that he was performing naked somersaults?”

There exist many erroneous fears about the toxicity of psychedelics. Whilst no drug is 100% safe, it is inexcusable when one comes across fellow medical colleagues whose knowledge and assumed fears of psychedelics are based wholly on media-driven preconceptions rather than evidence-based facts.

## Conclusion: A Return to Those Abused Children

We are today at a place in psychiatry where general medicine was 100 years ago. In the late nineteenth century patients were dying from infectious diseases, but the medical profession was yet to discover the miracle cure of antibiotics. We knew all about the epidemiology of smallpox, tuberculosis, and postoperative infections, but Victorian physicians were powerless to stop them. Then in the twentieth century antibiotics were discovered and this transformed the face of general medicine. Today in modern psychiatry we are similarly expert at the diagnosis, classification, and categorization of our common mental diseases. As psychiatrists, we write detailed manuals for the diagnosis and categorization of our common disorders: depression, anxiety and addictions. And we well-understand the common etiological roots of these disorders: childhood trauma. But our current best treatments are unsuccessful at effecting a lasting cure for trauma. Instead we provide a wide pharmacopeia of medications, from antidepressants to mood stabilizers to antipsychotics and hypnotics, which do little more than mask the symptoms of the underlying psychological problems.

Psychedelic therapies—and particularly MDMA-assisted psychotherapy for tackling underlying trauma—that aim not simply to paper symptomatically over the cracks of trauma, but rather provide an opportunity for patients for the first time in their lives to address and resolve those traumatic childhood memories, could be as transformative for twenty-first-century psychiatry as antibiotics were 100 years ago. We owe it to that population of patients—*my* patients suffering since childhood—to carry out this research.

## Electronic supplementary material

Below is the link to the electronic supplementary material.

ESM 1 **Required Author Forms** **Disclosure forms** provided by the authors are available with the online version of this article. (PDF 510 kb)
